# A chromosome-level reference genome assembly of the Reeve’s moray eel (*Gymnothorax reevesii*)

**DOI:** 10.1038/s41597-023-02394-7

**Published:** 2023-07-29

**Authors:** Kai Zhang, Yu Huang, Yuxuan Zhang, Rishen Liang, Qingqing Li, Ruihan Li, Xiaomeng Zhao, Chao Bian, Yongnan Chen, Jinhui Wu, Qiong Shi, Li Lin

**Affiliations:** 1 College of Animal Science and Technology, Zhongkai University of Agriculture and Engineering, Guangzhou, 510225 China; 2 Guangdong Provincial Water Environment and Aquatic Products Security Engineering Technology Research Center, Guangzhou, 510225 China; 3 Shenzhen Key Lab of Marine Genomics, Guangdong Provincial Key Lab of Molecular Breeding in Marine Economic Animals, BGI Academy of Marine Sciences, BGI Marine, Shenzhen, 518081 China; 4 Laboratory of Aquatic Genomics, College of Life Sciences and Oceanography, Shenzhen University, Shenzhen, 518060 China; 5 Agro-Tech Extension Center of Guangdong Province, Guangzhou, 510225 China

**Keywords:** Ichthyology, Comparative genomics

## Abstract

Due to potentially hostile behaviors and elusive habitats, moray eels (Muraenidae) as one group of apex predators in coral reefs all across the globe have not been well investigated. Here, we constructed a chromosome-level genome assembly for the representative Reeve’s moray eel (*Gymnothorax reevesii*). This haplotype genome assembly is 2.17 Gb in length, and 97.87% of the sequences are anchored into 21 chromosomes. It contains 56.34% repetitive sequences and 23,812 protein-coding genes, of which 96.77% are functionally annotated. This sequenced marine species in Anguilliformes makes a good complement to the genetic resource of eel genomes. It not only provides a genetic resource for in-depth studies of the Reeve’s moray eel, but also enables deep-going genomic comparisons among various eels.

## Background & Summary

Fish in family Muraenidae, commonly referred to as moray eels, are one group of the most species-rich of the order Anguilliformes. At present, approximately 210 species are recognized^1,2^. They are widely distributed in tropical, subtropical, and temperate waters worldwide, particularly in the region of Indo-Pacific^3–5^. Muraenidae species are cryptic, primarily found in shallow waters as rock and coral reef inhabitants, which play crucial roles in the maintenance of coral reef biodiversity^1^. They exhibit a wide range of color patterns, from uniform distinctive patterns of spots to blotches, bars, and reticulations. In a number of coastal countries, Muraenidae species are of commercial importance to the fishery industry due to their high nutritional value. Besides, their spectacular external coloration makes them well-liked in marine aquariums. To date, moray eels are not well-researched due to their cryptic habitats and irregularly aggressive behaviors^6^.

Anguilliformes species are scale-less with elongated bodies and usually lack pelvic fins^7–9^. Unlike other families in this order, some Muraenidae species don’t even have pectoral fins, and their opercular bones are degenerated with only small and round branchial pores opening outside^2^. Moray eels also have an unexpectedly wide variety of body sizes as per the whole length and mass. Their adults range in body mass from 4 to above 600 g, and in total length from 10 cm to 400 cm. Remarkably, the average number of vertebral numbers in morays varies by 2.4 folds, which is equal to the variance among extant snakes^10^. The maximum length and body shape are two parameters for diversifying among organisms, as each holds an effect on organismal-environment interactions^11,12^. Due to their distinctive morphological features, moray eels can be utilized as a good model for thoroughly exploring molecular mechanisms of evolutionary body patterns.

Both freshwater and marine eels differ a lot in phenotypic evolution, adaptation, and speciation and are closely related to chromosome evolution including rearrangements as has been proved by previous studies^13^. The majority of freshwater eels have only 19 pairs of chromosomes^14^, but some marine eels such as leopard moray eel (*Enchelycore pardalis*) and brown moray eel (*Gymnothorax unicolor*) have 21 pairs^15^. An in-depth investigation of this chromosomal difference between these eels may be instructive to reveal phenotypic, adaptive, and speciation differences between the two groups.

**Fig. 1:**
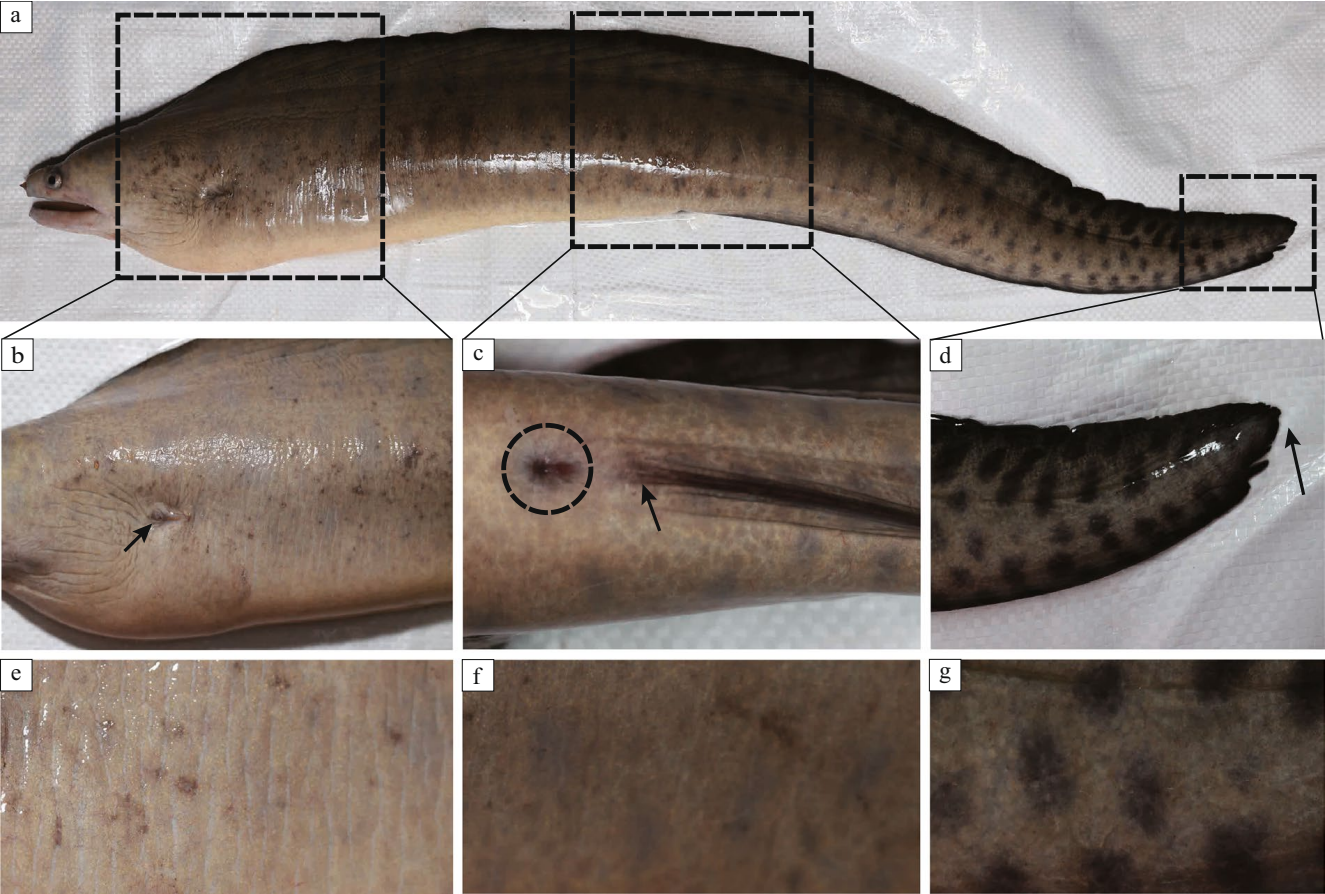
Images of the sequenced Reeve’s moray eel. (**a**) A lateral full-body view of the fish. (**b**) A thoracic side view of the fish. The arrow points to the gill apertures. The pelvic and pectoral fins are not visible in the field of this view. (**c**) A caudal ventral view of the fish. The dashed circle covers the cloacal area. The arrow points to where the caudal fin begins. (**d**) A lateral view of the fish’s tail. The caudal fin is not seen in the area pointed by the arrow. (**e**–**g**) An enlarged view of the thorax, ventral, and tail of the fish. Obviously, no scales are visible on the skin surface of these areas.

Previous studies have primarily concentrated on the morphology, taxonomy, and germplasm resource of moray eels, while limited genetic and genomic resources have largely constrained the conservation as well as utilization of these commercial species. Reeve’s moray eel (*Gymnothorax reevesii*), as a representative species in the family Muraenidae, has an elongated body and does not have pectoral and pelvic fins (Fig. 1). *G. reevesii* is a subtropical and tropical species in the Northwest Pacific, and most distribute from southern Japan to the South China Sea^16^. Its body color of yellowish brown to brownish, with numerous large obscure dark brownish spots in 3–5 rows along the body side. Among Muraenidae fishes, *G. reevesii* is a common catch in coastal areas of eastern and southern China with commercial importance to local fisheries^17^. In recent years it is also tamed as cultured fish and becomes one of the few Muraenidae species that can be artificially cultured with a vast developmental and economic potential. But until now very few studies have been focused on this species^18^, indicating that a huge research gap is still existed, and thereby further information about this species is needed to be explored. In our present study, a chromosome-level genome assembly of *G. reevesii* was constructed using an integrated strategy of MGI^19^ (second generation), PacBio (third generation), and Hi-C sequencing technologies, and we then characterized the high repeat content in the genome, inferred the phylogeny, and performed a chromosomal synteny analysis. This sequenced marine species in Anguilliformes makes a good complement to the genetic resource of eel genomes.

## Methods

### Sample collection

A female Reeve’s moray eel (Fig. 1a) was collected from Daya Bay Aquatic Testing Center in Huiyan District, Huizhou City, Guangdong Province, China. We pooled the muscle (about 5 g) of this individual for whole genome sequencing (short-read, long-read, and Hi-C sequencing), and the muscle, gonads, brain, and liver (50 mg for each tissue) for transcriptome sequencing. These samples were cut into small pieces and freshly frozen in liquid nitrogen, and then were stored at −80 °C until use. The Animal Ethics Committee of Zhongkai University of Agriculture and Engineering (Guangzhou, China) approved our sampling pipeline.

### DNA extraction and genome sequencing

Extraction and purification of genomic DNA (gDNA) from the muscle (2 g) was carried out using a blood & cell culture DNA kit (Qiagen, USA) in accordance with the manufacturer’s protocol.

The extracted gDNA (1.5 ug subsample) was randomly fragmented and used to create a 350-bp insert-size library by using MGIEasy universal DNA library prep set (MGI, China) for subsequent sequencing on a MGISEQ 2000 platform (MGI, China). A total of 65.39 Gb of paired-end raw reads (150 bp in length) were generated and then filtered via the SOAPfilter v2.2^20^ (default parameters) to remove adaptor sequences and low-quality reads. We finally obtained approximately 60.68 Gb of clean reads for estimation of the genome size and subsequent assembling.

Furthermore, we sampled 2 ug gDNA to construct long-read libraries by using a SMRTbell Express Template Prep Kit 2.0 for HiFi sequencing based on PacBio’s standard protocol (Pacific Biosciences, USA), which were sequenced through a PacBio Sequel II System. CCS software (SMRT Link v9.0)^21^ was then applied to generate the consensus sequences (-min-passes 1 --min-rq 0.99 --min-length 100). About 5.46 million consensus reads (68.88 Gb) with a mean length of 12.62 kb were obtained.

For the Hi-C sequencing, muscle tissue (about 1 g) from the same individual was collected, and DNA libraries were constructed by using GrandOmics Hi-C kit (the applied restriction enzyme is DpnII; GrandOmics, China) according to the manufacturer’s protocol. The Hi-C libraries were then sequenced on an Illumina Novaseq platform (Illumina, USA). In total, 305.18 Gb of Hi-C paired-end raw reads (150 bp in length) were generated. Subsequently, fastp^22^ was applied to filter the adaptors, and those reads shorter than 30 bp or of low-quality (quality scores < 20). Finally, 99.98% reads (304.92 Gb) were retained for construction of pseudo-chromosomes.

### RNA extraction and transcriptome sequencing

RNA samples were extracted from muscle, gonad, brain, and liver tissues (50 mg for each tissue) using a standard Trizol protocol (Invitrogen, USA), and purified using a Qiagen RNeasy mini kit (Qiagen, USA). RNA with equal amounts from each tissue was mixed for creating an Illumina cDNA library followed the manufacture’s guideline, which was then sequenced on a HiSeq X Ten platform (Illumina, USA). Around 6.01-Gb transcriptome data were generated for assistance to genome and gene annotations.

### Genome-size estimation

**Fig. 2:**
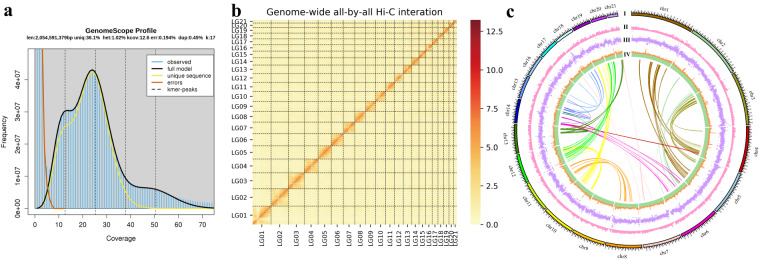
A chromosome-level genome assembly of the Reeve’s moray eel. (**a**) A GenomeScope k-mer plot. (**b**) A total of 21 distinct blocks were visualized in the Hi-C contact matrixes. (**c**) A Circos plot summarizing the genome features. From outside to inside: (I) the 21 pseudo-chromosomes, (II) gene density, (III) repeats, and (IV) GC content. Links inside the Circos refer to internal syntenic blocks among various chromosomes within the assembled genome.

To estimate the genome size of Reeve’s moray eel, a k-mer analysis^23^ was performed by using MGI clean reads. Through the k-mer counting (KMC) program^24^ and genome character estimator (GCE) software^25^, the 17-mer frequency was calculated. The genome size was then estimated by assessing the 17-mer depth distribution through the equation of G = K_num/K_depth (G is the genome size, K_depth represents the k-mer depth, and K_num stands for the total number of 17-mers). The estimated genome size of Reeve’s moray eel is therefore about 2.05 Gb, and the genomic heterozygosity rate was predicted to be 1.02% (Fig. 2a).

### *De novo* genome assembly

After obtaining subreads, the PacBio long reads were *de novo* assembled into contigs through hifiasm (v0.16.0)^26^ with default parameters. These contigs were then polished with Nextpolish (v1.10)^27^ using the MGI short reads to fix possible base errors. The primary genome assembly was 2.23 Gb in length, consistent with the estimated genome size.

**Fig. 3:**
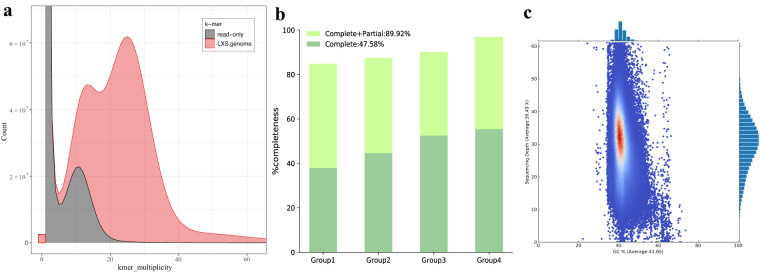
Quality assessment of the genome assembly of Reeve’s moray eel. (**a**) Merqury spectrum plot for the genome assembly. K-mers in the assembly (red) and only in the reads (gray) are presented. (**b**) Genome completeness assessment by CEGMA presenting the annotated core genes in the assembly. Green bars denote the complete genes, while light green bars denote the complete and partial genes combined. (**c**) GC-depth plot. The x- and y-axis represent GC content and sequencing depth respectively, and corresponding histograms are on the top and right.

We employed multiple methods to evaluate the quality of this assembly. First, Merqury v1.3^28^ shows that the estimated completeness, QV, and error rate of the assembly were 86.33%, 41.47 and 7.12e-05, respectively. A k-mer spectra plot is provided (Fig. 3a). Second, Benchmarking Universal Single Copy Orthologs (BUSCO) v5.2.2^29^ against actinopterygii_odb10 database was employed to assess the completeness of this genome assembly, showing that the assembly contains 94.89% of complete BUSCO genes including 88.3% single-copies and 6.59% duplicates (Table 1), suggesting that this genome assembly is of high quality. Third, Core Eukaryotic Gene Mapping Approach (CEGMA v2.5)^30^ also shows that 223 (89.92%) out of 248 core genes were successfully assembled (Fig. 3b). Finally, we mapped the long reads back to the assembly via minimap2 v2.15^31^, and calculated the average GC content and sequencing depth over 10-kb windows, which shows that the assembled genome is clean without contamination (Fig. 3c). Gfastats v1.3.1^32^ was applied to obtain assembly summary statistics (see more details in Table 1).

**Table 1:** Statistics of the assembled genome for the Reeve’s moray eel.

Genome assembly statistics	Data	
Total length	2,230,846,688 bp	
Number of contigs	579	
Largest contig length	118,230,600 bp	
Contig N50 length	53,970,335 bp	
Contig aUN	50,446,186 bp	
QV score	41.47	
Completeness	86.33%	
GC rate	41.61%	
Repeat elements	56.34%	
**BUSCO genome completeness score**	**Number**	**Ratio (%)**
Complete BUSCOs	3,454	94.89
Complete and single-copy BUSCOs	3,214	88.30
Complete and duplicated BUSCOs	240	6.59
Fragmented BUSCOs	66	1.81
Missing BUSCOs	120	3.30
Total number of Actinopterygii orthologs	3640	100

### Pseudo-chromosome construction

Based on this high-quality genome assembly, Hi-C technique was subsequently employed to construct pseudo-chromosomes for the Reeve’s moray eel. First, Hi-C clean reads were mapped to the assembled contigs using bowtie2 (v2.3.2)^33^ (-end-to-end --very-sensitive -L 30). Subsequently, HiC-Pro (v2.8.1)^34^ pipeline was applied to detect valid ligation products and only valid contact paired reads were retained for further analysis. Based on these valid reads, the primary assembly was oriented, ordered, and clustered onto chromosomes through LACHESIS^35^, with optimized parameters (CLUSTER_MIN_RE_SITES = 100, CLUSTER NONINFORMATIVE RATIO = 1.4, CLUSTER_MAX_LINK_DENSITY = 2.5, ORDER MIN N RES IN SHREDS = 60, ORDER MIN N RES IN TRUNK = 60). JuiceBox v1.11.08^36^ was employed for manual correction of placement and orientation mistakes with glaring distinct chromatin interaction patterns. We hence obtained the final genome assembly with a size of 2.17 Gb, of which 97.87% are anchored into 21 chromosomes (Fig. 2). The scaffold and contig N50 values of the overall chromosome-level genome assembly are 112.89 Mb and 53.38 Mb, respectively, reaching a relatively high level among sequenced fish species.

### High repeat content in the Reeve’s moray eel genome

Transposable elements (TE) in the *G. reevesii* genome were predicted by combination of homology-based and *ab initio* predictions. In general, default settings from RepeatModeler^37^ and MITE-Hunter^38^ were employed to obtain an *ab initio* repetition library, which was then aligned to Repbase^39^ with TEclass tool^40^ for classifying the details of each repeat family. By mapping sequences against the Repbase TE library and *de novo* repeat library, RepeatMasker^37^ was applied to check known and new TEs so as to further identify these repeats across the assembled genome. Finally, a total of 1.23 Gb of repetitive sequences were annotated in the assembly, including 1.09 Gb of TEs (Table 2).

**Table 2:** Repetitive sequences in the genome of Reeve’s moray eel.

Type		Number	Length	% of the genome
Transposable elements	LINE	540,403	165,143,295	7.59
	SINE	144,639	20,595,693	0.95
	LTR	610,545	200,035,359	9.20
	DNA	2,519,498	560,924,285	25.80
	Helitron	199,516	45,273,868	2.08
	MITE	314,975	98,390,223	4.52
	Total	4,329,576	1,090,362,723	50.15
Tandem Repeats		232,895	37,337,393	1.72
Simple repeats		36,791	5,686,723	0.26
Low complexity		3,202	612,494	0.03
Unknown		442,768	85,007,759	3.91
Other		30,549	6,001,429	0.28
Total Repeats		5,075,781	1,225,008,521	56.34

**Fig. 4:**
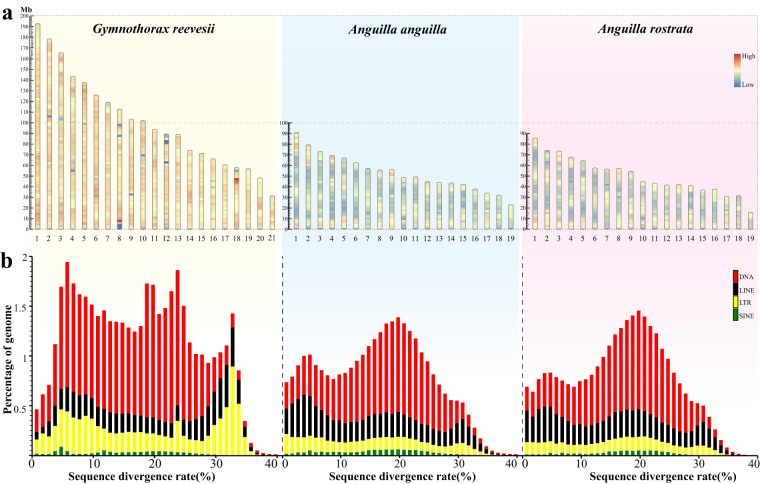
The Reeve’s moray eel had a higher content of repetitive sequences than freshwater eels. (**a**) Repeat density throughout the chromosomes of the examined eels. The redder indicates the higher repeat content, while the bluer marks the lower repeat content. (**b**) Distribution of the four major TE types. Red, black, yellow, and green bars represent DNA, LINE, LTR, and SINE repeats, respectively.

In summary, repetitive sequences account for up to 56.34% of the genome of Reeve’s moray eel, including 50.15% of TEs (Table 2), which content is much higher than those of freshwater eels^14^ and most sequenced fishes so far that usually have a TE content less than 45%^41^. We then detected the repeat content in each chromosome of the Reeve’s moray eel and the freshwater eels. A plot of the repeat distribution throughout corresponding chromosomes, drawn by the RIdeogram package^42^, indicated that each chromosome of the Reeve’s moray eel also has a higher repeat content when compared to those freshwater counterparts (Fig. 4a).

More specifically, the Reeve’s moray eel genome has 50.15% of TEs, accounting for 89.01% of the total repetitive sequences. The combined length of the sequences representing each type of DNA, LTR, LINE, and SINE are 560.92, 200.04, 165.14, and 20.60 kb, respectively, which shows remarkable expansions of DNA and LTR sequences in the genome of Reeve’s moray eel compared to the freshwater eels (Fig. 4b). Considering that all these eel genomes with Mb-level contig N50 values were assembled from long reads with sufficient coverage, this difference appears be real among genomes and not the result of unassembled repeats in the freshwater eel genomes.

### Gene annotation and functional assignment

In the repeat-masked genome, gene prediction was carried out using three methods, including homology, transcriptome-based, and *ab initio* annotations. For the homology prediction, GeMoMa^43^ was employed to align homologous proteins from relevant species (including Japanese eel *Anguilla japonica*, spotted gar *Lepisosteus oculatus*, zebrafish *Danio rerio*, three-spined stickleback *Gasterosteus aculeatus*, and Fugu *Takifugu rubripes*) to our assembly to predict gene structures. For the transcriptome-based gene prediction, clean transcriptome reads were mapped to the assembled genome by STAR^44^. PASA^45^ was then applied to predict open reading frames (ORFs) and stringtie^46^ was employed to assemble transcripts for gene structure annotation. For the *ab initio* annotation, transcriptome reads were *de novo* assembled by Trinity (v2.13.2)^47^, and 300 full-length transcripts were randomly selected to build a library as a training set. Based on this training set, Augustus^48^ with default parameters was then employed to predict genes. Finally, EVidenceModeler (EVM)^45^ was applied to create a combined and non-redundant gene set after removal of those miscoded genes, and the final protein sequences were deduced by standard genetic codes.

Motifs/domains and gene functions of these annotated genes were predicted by blasting (BLASTp) the deduced protein sequences against various public databases, including SwissProt^49^, NCBI NR, KEGG^50^, KOG^51^, and Gene Ontology^52^ (GO), with an E-value cutoff of 1e−05.

Finally, in the assembled genome of Reeve’s moray eel, a total of 23,812 protein-coding genes were annotated, with an average gene length of 36,705.61 bp, an average coding sequence (CDS) length of 1,738.42 bp, and an average number of 8.51 exons per gene. Among these genes, 96.77% (23,812 genes) were annotated in the searched protein databases (Table 3). In addition, the CDSs were further translated into protein sequences with standard genetic codes, followed by self-alignment using BLASTp for construction of internal syntenic blocks by MCscan^53^ with parameters “-a -e 1e-5 -u 1 -s 8”. A Circos^54^ plot (Fig. 2c) was then generated to show (I) lengths of 21 pseudo-chromosomes, (II) gene density (percentage of genes per 100-kb window), (III) repeat density (minimum 0%, maximum 100%), (IV) GC content (minimum 30%, maximum 65%), and internal syntenic blocks (in the center).

**Table 3:** Gene structures and function annotation.

Item	Number	Average length (bp)
Gene	23,812	36,705.61
Exon	10.07 (per gene)	172.57
Intron	—	3,853.67
**Database**	**Number**	**Percentage (%)**
Swissprot	21,479	90.20
KEGG	16,154	6.84
KOG	15,569	65.38
GO	14,997	62.98
NR	22,154	93.04
All	23,044	96.77

### Phylogenetic tree

**Fig. 5:**
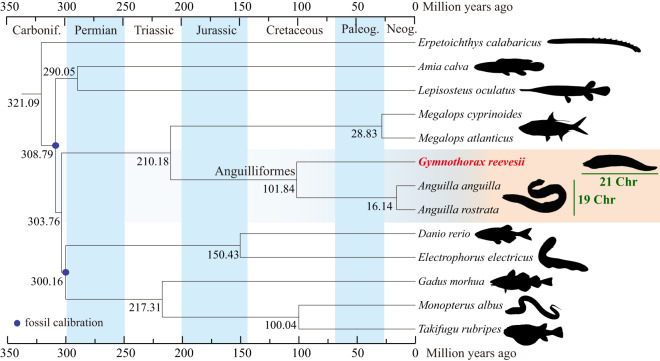
A phylogenetic tree of *G. reevesii* and other representative ray-finned fish species. Number at each node means the estimated divergence time. Blue dots denote the fossils used for time calibration. The Anguilliformes lineage is highlighted in a yellow shadow. The pair numbers of chromosomes are 21 and 19 for the Reeve’s moray eel and freshwater eels, respectively.

A species tree was constructed using those single-copy orthologs from the whole genomes of Reeve’s moray eel and other 12 representative ray-finned species, with ropefish (*Erpetoichthys calabaricus*) as the outgroup. Both the Maximum Likelihood tree (constructed using PhyML^55^) and the Bayesian tree (inferred by MrBayes^56^) present the same topology with 100% for the node bootstrap values or 1 for the node support values. The consensus tree was further time-calibrated using MCMCTREE program in the PAML package^57^. The tree shows that Reeve’s moray eel and two examined freshwater eels make up the Anguilliformes clade, forming a sister group to the Elopiformes composed of two tarpons, and that Reeve’s moray eel diverged from both freshwater eels 101.84 million years ago (Mya; Fig. 5). These data are consistent with previous reports^58–60^.

## Data Records

The genome assembly and raw reads of the genome and transcriptome sequencing for Reeve’s moray eel were deposited at NCBI under the accession number PRJNA934055^61^. Raw reads are available in the Sequence Reads Archive (SRA) with the accession number SRP430070. The genome assembly was deposited at GenBank with the accession number GCA_029721435.1^62^. Repeat and gene annotation files are publicly available in FigShare depository with accessions 10.6084/m9.figshare.22357987^63^ and 10.6084/m9.figshare.22358209^64^, respectively.

## Technical Validation

Quality of extracted gDNA was detected by agarose gel electrophoresis (a main band at 20 kb) and Nanodrop spectrophotometer (Thermo Fisher Scientific, USA). The quality of the isolation met the standards that OD260/280 value ranges from 1.8 to 2.0, and OD260/230 is between 2.0–2.2. Quality of extracted RNA was examined by a 2100 Bioanalyzer (RIN > 7.0, 28 S/18 S > 1.0; Agilent Technologies, USA).

**Fig. 6:**
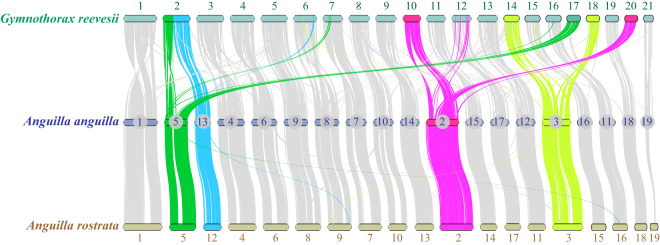
A synteny analysis of the chromosomes between Reeve’s moray eel and freshwater eels. Chromosomes are ordered from the longest to the shortest. Major reorganizations between Reeve’s moray eel and the freshwater eels are colorized.

Reeve’s moray eel has 21 pairs of chromosomes (Chr) whereas most freshwater eels have only 19 pairs, which is evidenced by both karyotypic observation and genomic sequencing^14,65–67^. We therefore performed a chromosomal synteny analysis using the JCVI package^68^. Our plot shows that European eel and American eel had perfect one-to-one correspondences among their chromosomes (Fig. 5), and Reeve’s moray eel showed a generally conserved chromosomal synteny although with a few large fragmental exchanges. For example, both Chr2 and Chr3 in American eel and European eel correspond to two chromosomes (10 & 20, and 14 & 18, respectively) of Reeve’s moray eel. In addition, part of the Chr5 fused to the end of the Chr13 (American eel) or Chr12 (European eel) of the freshwater eels, forming Chr2 in the Reeve’s moray eel, and the other half of the Chr5 was preserved as a single chromosome (Chr17) in moray eel, presenting a two-to-two synteny pattern (Fig. 6). The chromosome synteny analysis implies that there is generally conserved chromosomal synteny between moray eel and freshwater eels, but a few fissions and fusions happened after their divergence, leading to 21 and 19 pairs of chromosomes in the two lineages respectively^14^.

## Data Availability

The versions and parameters of bioinformatic tools used in this study have been described in the Method section. If no parameter is provided, the default is used. No custom code was used.
